# Use of Screen Media and Mental Health: Effects on Adolescents and Pre-adolescents

**DOI:** 10.31729/jnma.5518

**Published:** 2021-09-30

**Authors:** Bibek Adhikari

**Affiliations:** 1 Nobel Medical College and Teaching Hospital, Kanchanbari, Biratnagar, Morang, Nepal

**Keywords:** *adolescent*, *mental health*, *screen time*

## Abstract

The children and adolescents of today's generation are growing up in a media-saturated world. Digital media use has become the most common sedentary leisure time activity among children and adolescents. In the past decade, the development of mobile and digital technologies has taken place at such a rapid rate that researchers have had difficulties reporting evidence within limited timeframes. Growing evidence indicates that screen media usage by teenagers and pre-adolescents has deleterious ramifications on mental wellbeing. In addition to the benefits of screen media for exposure to a wide range of information and quick communication, the use of screen media has been correlated with adverse physical, psychological and social health consequences. This study focuses on the increasing use of screen media and their consequences on the various aspects of mental health of adolescents and preadolescents.

## INTRODUCTION

Digital media use covers screen-based activities such as internet browsing, computer use, smartphone use, television viewing and playing video games.^1^ Using portable devices such as smartphones and laptops has made use of media available 24 hours a day. There is growing evidence that use of digital media may play a role in cognition i.e. brain processes involved in knowledge, intellect and action and academic success in children and adolescents. Along with the benefits of screen media to reach a wide range of information and easy communication, use of screen media has been correlated with detrimental physical, psychological and social health effects.

## EVIDENCE FROM RECENT STUDIES

Recent empirical research has indicated that use of screen media can decrease functional connectivity between cognitive areas.^1^ According to a longitudinal study and meta-analysis of 58 cross-sectional studies performed by Renauetal (2019),^2^ television viewing and video game playing but not overall screen media were inversely correlated with the academic success of children and adolescents. One study found that adolescents who spent more than 7 hours a day on overall electronic media were 40% less likely to achieve good academic success, and those who spent 2 to 4 hours a day were 1.23 times more likely to achieve outstanding grades than those who spent less than 2 hours a day.^3^ It has been found that prolonged television watching time in children reduces concentration and cognitive functioning and raises behavioral disorders and poor eating patterns, which can affect academic performance. Previous studies have shown that playing video games is inversely correlated with emotional and mental health which triggers psychological and behavioral issues.^4,5^

In a retrospective longitudinal cohort study of adolescents aged 15 and 16 years and without Attention-Deficit / Hyperactivity Disorder (ADHD) symptoms, there was a strong correlation between higher prevalence of modern digital media use and associated ADHD symptoms during a 24-month follow-up.^6^ In addition, a longitudinal study conducted by Boers et al. among 3826 adolescents found a within person association, based on repeated measures, between social media and television use with symptoms of depression in adolescence.^7^ Depression is associated with severe academic, psychosocial and cognitive disability during adolescence development. Mental health disorders, including depression, are expected to be among the leading causes of morbidity and mortality among adolescents by 2020.^8^ Researchers have attributed this increase in internalizing problems to the amount of time children spend in front of digital screens.

Many studies found significant correlations between screen time and depression factors including selfesteem and isolation. Depression is correlated with 4 different forms of screen time mainly social media, television, online games, and computer use. Three Media effect theories also known as displacement hypothesis, upward social comparison and reinforcing spirals have been commonly used to explore correlations between screen time and mental health. Displacement hypothesis states that all screen time has detrimental effects on mental well-being, because it displaces time spent on healthy activities for instance Physical exercise.^9,10^ Further, upward social comparison indicates that the impact of the screen time on mental health depends on the nature of the content. Upward social comparison happens when people compare themselves with others in a more favorable position,^11,12^ especially those with perfect bodies and lives. Exposure of television depicting idealized bodies has been shown to lead to reduced body satisfaction, which in turn results in more serious depressive symptoms. Social upward comparison has also been found when using social media. Finally, reinforcing spirals suggests that the effects of screen time are influenced by content.^13^ It adds that people look for and select information that is compatible with their cognitions. Reinforcing spirals has been documented for exposure to aggressive content and violence like in violent movies and on political information and political attitudes. It might be especially important for screen time which features algorithm based content feeding that is repeated inside a closed system for example a filter bubble.^14^ Within a filter bubble, algorithms automatically suggest content based on past search and selection actions a person is likely to be interested in.

Boers E, et al.,^7^ in their study found that elevated mean levels of social media use over 4 years and a further rise in social media usage over the same year is correlated with increased depression. They also found that a tendency to indulge in high mean levels of television use over 4 years has been correlated with less depression. However any further rise in television use in the same year was correlated with increased depression. In addition, they found that high levels of computer use over 4 years was correlated with increased depression; however, any further rise in computer use over the same year is not correlated with "increased" depression. Lastly, post-hoc analysis shows that self-esteem but not exercise is correlated with depression in adolescence, and that only social media and television have a time-varying negative correlation with self-esteem within person association.

Next area of health concern with the children's and adolescents' media use is its effect on sleep. Concerns about media use and sleep include the fact that the use of media at or near bedtime can result in fewer hours of sleep and poor quality of sleep. Concerns have been raised about the elevated use of media leading to daytime sleepiness, which could affect academic performance for children and produce other physical health problems. According to American Academy of Sleep Medicine, children 6-12 years of age should get 9 to 12 hours of sleep and teens 13-18 years should get 8 to 10 hours each night. Sleeping the required hours on a daily basis is important and correlated with better health outcomes, including enhanced concentration, behavior, understanding, memory, cognitive control, quality of life and mental and physical wellbeing. Carter B, et al.^15^conducted meta-analysis which included 20 previous studies from around the globe; in total the studies included 125,198 children. The analysis found evidence regarding the relationship between media and sleep. At first, researchers observed a strong and consistent association between using media before bedtime and reduced amount of hours of sleep. The meta-analysis showed that the probability of not getting enough sleep was 2 times higher for children and adolescents who used the media before bedtime. Furthermore, having access to a media device in a sleeping environment, even if it was not actively being used near bedtime, was associated with a lack of sleep. Children who used devices near bedtime were also more likely to have poor sleep quality. Researchers have found that even getting access to a media device at bedtime is correlated with reduced sleep quality. Also, getting access to media at bedtime as well as using them near-bedtime media is associated with increased sleepiness during the day. The American Academy of Pediatrics recommends that children should not sleep with devices in their bedrooms, including televisions, computers, smartphones and tablets. Results reported by Ra CK, et al.^6^ affirm the 2016 recommendations of the American Pediatrics Association to emphasize practices that promote the executive functioning and well-being of adolescents, including sleep, physical exercise, distraction free homework and meaningful interaction with family and friends.

## WAYS FORWARD

Adolescents and children's social media and entertainment use should be monitored to prevent the development of depression and the exacerbation of existing symptoms over time. Education and public health experts should consider monitoring and reducing the use of screen media as methods to enhance the academic performance of children and adolescents. In conclusion, strategies to minimize device accessibility and usage need to be established and evaluated. Intervention should include a multidisciplinary approach by teachers and health professionals to empower parents to minimize detrimental effects on the health of children and adolescents.
